# Protein Kinase C δ: a Gatekeeper of Immune Homeostasis

**DOI:** 10.1007/s10875-016-0323-0

**Published:** 2016-08-19

**Authors:** Elisabeth Salzer, Elisangela Santos-Valente, Bärbel Keller, Klaus Warnatz, Kaan Boztug

**Affiliations:** 1 CeMM Research Center for Molecular Medicine of the Austrian Academy of Sciences, Lazarettgasse 14 AKH BT 25.3, Vienna, Austria; 2 Center for Chronic Immunodeficiency, University Medical Center Freiburg and University of Freiburg, Freiburg, Germany; 3 Department of Pediatrics and Adolescent Medicine, Medical University of Vienna, Lazarettgasse 14 AKH BT 25.3, Vienna, Austria; 4 Ludwig Boltzmann Institute for Rare and Undiagnosed Diseases and CeRUD Vienna Center for Rare and Undiagnosed Diseases, Vienna, Austria

**Keywords:** PRKCD, systemic lupus erythematosus, autoimmunity, immunodeficiency

## Abstract

Human autoimmune disorders present in various forms and are associated with a life-long burden of high morbidity and mortality. Many different circumstances lead to the loss of immune tolerance and often the origin is suspected to be multifactorial. Recently, patients with autosomal recessive mutations in *PRKCD* encoding protein kinase c delta (PKCδ) have been identified, representing a monogenic prototype for one of the most prominent forms of humoral systemic autoimmune diseases, systemic lupus erythematosus (SLE). PKCδ is a signaling kinase with multiple downstream target proteins and with functions in various signaling pathways. Interestingly, mouse models have indicated a special role of the ubiquitously expressed protein in the control of B-cell tolerance revealed by the severe autoimmunity in *Prkcd*^−/−^ knockout mice as the major phenotype. As such, the study of PKCδ deficiency in humans has tremendous potential in enhancing our knowledge on the mechanisms of B-cell tolerance.

## Introduction

Protein kinase C δ (PKCδ) is an essential regulator of peripheral B-cell development and a critical regulator of immune homeostasis [1, 2]. The protein was discovered in 1986 [3, 4], and several studies have addressed its structure and biological functions [5–13]. Complex activation patterns [5, 7–9, 13, 14] and opposing PKCδ functions depending on the activating stimuli and the investigated model system have been described [8, 11, 12].

**Fig. 1:**
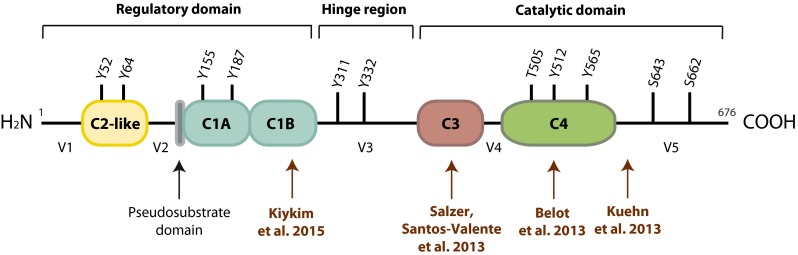
PKCδ structure and domains. This figure shows structural domains and phosphorylation sites on PKCδ as well as the localization of important sequences and the hitherto described *PRKCD* mutations in humans (modified from atlasgeneticsoncology.org/Genes/GC_PRKCD.html)

**Fig. 2:**
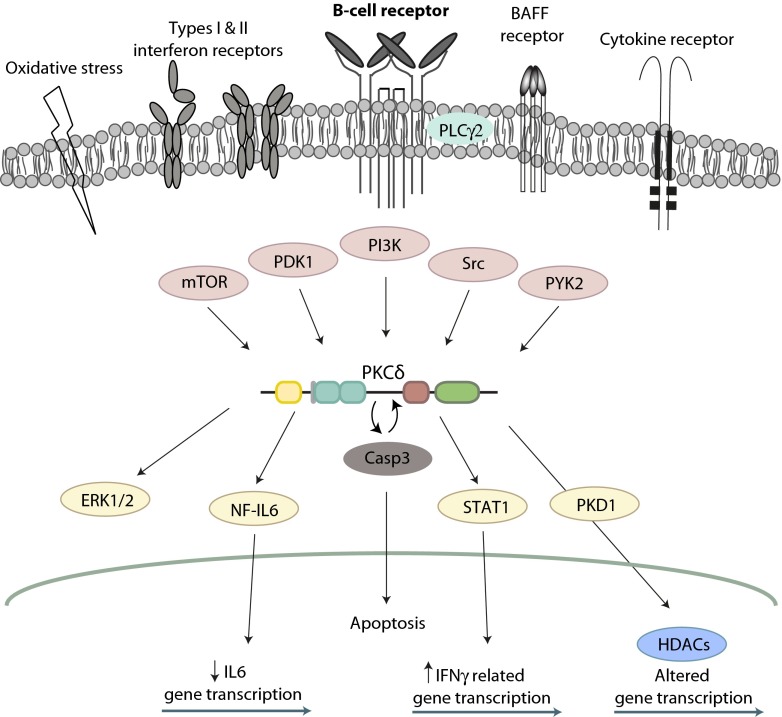
Overview on PKCδ signaling. This figure provides a basic overview of the receptors and molecules involved in PKCδ activation and of the described PKCδ activating and inhibitory roles following a variety of stimuli. Functions of PKCδ in mitochondria are not depicted. More details are outlined in the text. Structures depicted in *pink* represent upstream and those depicted in *yellow* represent downstream components involved in PKCδ activation. *mTOR*: mechanistic target of rapamycin; *PDK1*: phosphoinositide-dependent kinase-1; *PI3K*: phosphoinositide 3-kinase; *PYK2*: protein tyrosine kinase 2; *ERK1/2*: extracellular signal-regulated kinases 1 and 2; *NF-IL6*: nuclear factor of interleukin 6; *Casp3*: caspase 3; *STAT1*: signal transducer and activator of transcription 1; *IFNγ*: interferon gamma; *PKD1*: protein kinase D isoform 1; *HDACs*: histone deacetylases

PKCδ is ubiquitously expressed and activated in response to a broad variety of stimuli. In response to specific stimuli, several tyrosine residues in PKCδ can be phosphorylated by different tyrosine kinases (Fig. 1), leading to individual phosphorylation patterns and possibly differential activation of downstream targets. Among its main roles, PKCδ is responsible for the regulation of survival, proliferation, and apoptosis in a variety of cells including lymphocytes (reviewed in [15]) (Fig. 2).

In 2002, *Prkcd* knockout mice [1, 2] revealed an essential role for this kinase in B-cell homeostasis and tolerance. Due to defective negative selection in germinal centers and autonomous B-cell hyperproliferation in the periphery [2], autoreactive B cells accumulate in these mice. Consequently, *Prkcd* knockout mice develop systemic autoimmunity evidenced by autoantibodies, immune complex-mediated glomerulonephritis, lymphadenopathy, splenomegaly, and show B-cell infiltration in several organs and tissues [2].

Autoimmune phenomena arise when mechanisms preventing immune responses directed against the self-antigens are impaired [16]. Primary immunodeficiencies (PIDs) are caused by inborn defects in different arms of the immune system and have been described to result in autoimmune manifestations [17]. The clinical phenotype of autoimmune disorders varies greatly depending on the etiology and target organs. Systemic lupus erythematosus (SLE) is a heterogeneous, complex, and multifactorial autoimmune disease caused by defects in innate and adaptive immunity [18] and is characterized by a multifactorial loss of immune tolerance [16]. Given the phenotype of *Prkcd* knockout mice, a potential role of PKCδ in the pathogenesis of SLE has been proposed.

The effect of germline mutations affecting PKCδ in humans and its link to systemic autoimmunity had remained elusive until 2013, when we and others identified human PKCδ deficiency as a novel PID with severe SLE-like autoimmunity [19–21]. The following review provides a detailed summary of PKCδ protein structure and functions and human PKCδ deficiency.

## PKCδ Structure, Activation, Regulation, and Role in Apoptosis

The family of protein kinase C (PKC) contains serine/threonine kinases that execute key roles in various cellular processes, including cell proliferation, apoptosis, and differentiation [22]. PKCδ is a 78 kDa and 676 residues long protein included in the group called novel PKCs [3, 4, 23]. It is encoded by the *PRKCD* gene localized in chromosome 3 in humans and in chromosome 14 in mice [24]. The kinase is structurally divided into a regulatory and a catalytic domain that contains four constant (C) and five variable (V) regions. The variable region 3 (V3), also called hinge region, separates the catalytic and regulatory domains of the protein (reviewed in [15]) (Fig. 1).

The catalytic domain of PKCδ is required for enzyme activity and includes the C3 and C4 domains, encompassing ATP- and substrate-binding sequences, respectively (reviewed in [15]). The regulatory domain contains two constant regions (C1- and C2-like) and a pseudosubstrate. The C1 domain of PKCδ enables its binding to membranes as it contains hydrophobic residues that bind diacylglycerol (DAG) and phorbol 12-myristate 13-acetate (PMA) (reviewed in [6]). As a novel PKC, PKCδ is a calcium-independent, phospholipid-dependent kinase containing a C2-like domain, which lacks essential residues that allow conventional PKCs to bind Ca^2+^ [10, 15]. Situated between the C1- and C2-like domains, the pseudosubstrate keeps PKCδ in an inactive folded conformation, blocking access to the substrate-binding pocket (reviewed in [15, 25]).

A broad variety of stimuli lead to PKCδ activation through phosphorylation of serine/threonine and tyrosine residues, as well as proteolytic cleavage into an active fragment [14, 23]. Full kinase activity requires autophosphorylation of Ser-643 (turn motif), phosphorylation of Ser-662 (hydrophobic region) mediated by PKCζ or mTOR (mechanistic target of rapamycin), and Thr-505 phosphorylation by PDK1 [8, 9, 13, 14] (Figs. 1 and 2).

Protein activity is also regulated by the phosphorylation of specific tyrosine residues according to the stimuli employed (reviewed in [26]) (Fig. 1). In the hinge region, phosphorylation of Tyr311 and Tyr332 in response to apoptotic agents enables caspase 3 to cleave PKCδ. The proteolytic cleavage of PKCδ by caspase 3 generates a 40 kDa catalytic active fragment capable of translocating to mitochondria and/or nucleus [6, 14, 27] and promoting apoptosis [28, 29]. Apart from conformational changes after binding of DAG and autophosphorylation, activation of PKCδ depends on Ser/Thr phosphorylation by PDK1 [30]. Also, Src kinase family members, PYK2 as well as growth factor receptors phosphorylate tyrosine residues of PKCδ and modulate its enzymatic activity [23, 31] (Fig. 2).

In contrast to most other PKC isoforms, PKCδ conveys proapoptotic signals upon a variety of stimuli not only through nuclear but also mitochondrial and cytosolic pathways [32]. When activated, PKCδ translocates to the nucleus [33] and, possibly through JNK signals, promotes proximal regulation of apoptosis through cytochrome C release, PARP cleavage, histone phosphorylation [34], and caspase 3 activation. Nuclear retention can be prolonged and intensified by generation of the catalytic fragment of PKCδ by caspase 3 [33]. Engagement of apoptosis via the mitochondrial pathway has been described in response to the diacylglycerol analog PMA [22]. In brief, PKCδ phosphorylates phospholipid scramblase 3 (PLS3) at the mitochondrial site, subsequently facilitating mitochondrial targeting of tBid and apoptosis induction [35].

Moreover, in addition to the membrane-bound form of PKCδ, whose phosphorylation is regulated by DAG accumulation and membrane translocation upon PMA, a tyrosine-phosphorylated form of PKCδ is also found in the soluble fraction of cells in response to oxidant stress and display distinct substrate specificity. This PKCδ form is phosphorylated by Src family kinases and possesses lipid-independent catalytic functions, being able to phosphorylate substrates not only on lipid membranes but also in several compartments of the cell [36] (reviewed in [14]). Description of this lipid-independent kinase provides an explanation to PKC-dependent phosphorylation of substrates outside membrane compartments such as myofibrillar proteins in sarcomeres, structures not associated with lipid membranes [37] (reviewed in [14]).

Although numerous studies have tried to elucidate PKCδ activation and biological functions, its complex activation patterns and roles in various pathways remains incompletely understood.

## PKCδ Signaling and Functions in Lymphocytes

In B cells, recent evidence suggests a PKCδ-, RASGRP-, and calcium-dependent ERK signaling as a critical proapoptotic pathway promoting negative selection [38] (Fig. 2). This pathway is biochemically distinct from DAG-induced ERK activation. Moreover, PKCδ phosphorylation and activity plays a central role in the BCR-signalosome-independent, IL-4-dependent activation of B cells [39], adding another level of complexity to the regulation of survival, proliferation, and apoptosis by PKCδ.

After binding of B-cell activating factor (BAFF) to its receptor and after B-cell receptor (BCR) stimulation, PKCδ is phosphorylated at Thr505 [38, 40, 41] involving activation of the PI3K [41]. In the absence of PKCδ, both tonic (ligand-independent) and antigen receptor-induced BCR signaling is increased [38]. Additionally, a PI3K-/PKCβ-independent alternative pathway downstream of the BCR is induced after prestimulation of B cells with IL-4 [42]. In contrast to the classical signalosome-dependent pathway, it requires the direct interaction of LYN, inducing phosphorylation of PKCδ at Tyr311 and resulting in increased phosphorylation of protein kinase D (PKD) [39]. Downstream of PKD, histone deacetylases (HDAC) 5 and 7 are phosphorylated and excluded from the nucleus, thereby facilitating the transcription of genes, which are repressed by these HDACs [39]. The specific targets of these class IIa HDACs in B lymphocytes have not been investigated in detail but include MEF2-dependent promoters [43] playing a substantial role in B-cell proliferation and survival [44]. In vitro studies demonstrated the interference of HDAC inhibition with plasmablast differentiation [45]. HDACs may also be involved downstream of PKCδ in the expression of CIITA which regulates MHC class II expression [46] (Fig. 2).

In T cells, PKCδ has a negative role in TCR/CD3-mediated IL-2 production and in T-cell proliferation with consequent increased signaling responses in PKCδ^−/−^ T cells [47]. Its importance to ERK pathway signaling in T cells has also been demonstrated as lack of T-cell PKCδ activity prompts reduced ERK signaling [48].

Until now, critical roles for PKCδ in regulation of survival, proliferation, and apoptosis, mainly of B cells, have been recognized, although more comprehensive studies are still necessary to fully elucidate its complex functions in lymphocytes.

## PKCδ Deficiency in Mice

Differentiation and development of lymphocytes in the bone marrow of Prkcd-deficient mice was reported normal [49]. Also, peripheral T- and NK-cell development seemed unaffected [49]. However, in peripheral blood and secondary lymphoid organs, these mice showed an increased frequency of mature B cells, which could be attributed to autocrine IL-6 driven proliferation [1, 2], since PKCδ negatively interferes with the production of this cytokine through phosphorylation of the nuclear factor (NF)-IL6 (2) (Fig. 2).

In consequence, mice with mutations in *Prkcd* develop severe humoral autoimmunity, marked by autoantibodies, immune complex-mediated glomerulonephritis, lymphadenopathy, splenomegaly, and B cell infiltrations in kidney, liver, lung, and salivary glands, respectively [2]. In a hen-egg-lysozyme transgenic mouse model, it has been shown that the induction of anergy and thus peripheral tolerance toward soluble self-antigens was impaired, while the deletion of antigen-specific B cells by membrane-bound self-antigens was not affected in the absence of PKCδ [1]. More recently, Limnander et al. were able to elucidate impaired BCR-induced apoptosis at the T1 B cell stage as the major mechanism contributing to defective peripheral tolerance and overt autoimmunity in the absence of PKCδ [38].

Transgenic mice expressing doxycycline-induced dominant negative PKCδ in T cells show reduced ERK pathway signaling, decreased expression of *Dnmt1*, and increased expression of methylation-sensitive genes. Such mice presented with lupus-like autoimmunity including anti-double-stranded DNA (dsDNA) antibodies and immune complex glomerulonephritis [48]. Thus, PKCδ deficiency is associated with a loss of tolerance in peripheral B-cell development and possibly also increased T-cell activation, causing systemic autoimmunity in these mice [1, 2, 48].

In brief, studies in PKCδ-deficient mice revealed non-redundant roles for this kinase in B-cell proliferation and peripheral tolerance as well as in T-cell activation.

## PKCδ in Human Systemic Lupus Erythematosus

Systemic lupus erythematosus is mainly an immune complex-mediated disease characterized by the presence of multiple autoantibodies including antinuclear antibodies (ANAs). The loss of immune tolerance in SLE is multifactorial and includes a breakdown of T- and B-cell tolerance [16]. Given the phenotype of *Prkcd* knockout mice, a potential role of PKCδ in the pathogenesis of SLE could be proposed. Gorelik et al. [48, 50, 51] postulated that inhibition of PKCδ and disturbed ERK signaling in T cells are involved in the development of autoimmunity in active SLE. Reduction of PKCδ phosphorylation at Thr505 due to oxidative stress and nitration of PKCδ prompts decreased ERK signaling in T cells of SLE patients, which leads to reduced DNMT1 activity and hypomethylation of regulatory sequences of sensitive genes such as *LFA1* (encoding CD11a) and *TNFSF7* (encoding CD70), therefore causing T-cell activation and contributing to T-cell autoimmunity. This model was corroborated by the occurrence of drug-induced lupus by demethylating drugs like hydralazine and others [52].

Interestingly, comparative network analysis of the phosphoproteomes of peripheral blood mononuclear cells of SLE patients versus healthy controls described PKCδ (beside Src kinases, the NF-kB signaling component RelA and HDAC1) as one of the genes with the most connections to other proteins in the altered network of SLE patients and therefore with significant role in the network stability [53].

In addition, a link between IFN signaling and PKCδ was established by the association of PKCδ activity with STAT1 activation through Ser727 phosphorylation, which is essential for the expression of IFN response genes [54, 55] (Fig. 2). Huang et al. demonstrated that IFN-alpha-induced expression of IFIT4 requires activation of PKCδ and JNK, as well as STAT1 phosphorylation at Ser727 [56]. Thus, a decreased activity of PKCδ in SLE monocytes may contribute to the proinflammatory effect ascribed to IL-10 in SLE [57]. However, in conditions with elevated levels of IFN type I cytokines such as during infections or in autoimmune diseases, PKCδ shows opposite activity, suppressing IL-10 activation of STAT1 through tyrosine phosphorylation [57].

Given the multiple activities of PKCδ, among others in T-cell activation and IFN signaling, it is not surprising that an altered PKCδ function contributes, probably through several mechanisms, to the complex autoimmunity observed in SLE.

## Human PKCδ Deficiency: Genetics and Clinical Phenotype

In our previous work, we identified a patient (P1) with an autosomal recessive disorder caused by loss-of-function splice-site mutation in *PRKCD* (c.1352 + 1G > A) (Fig. 1), within the catalytic domain of the protein, whose clinical picture included antibody deficiency with respiratory tract infections from the first year of life and immune dysregulation reminiscent of a CVID-like disease [21]. Partial clinical improvement could be achieved with the initiation of immunoglobulin G substitution at the age of 4 years. Features of immune dysregulation, including autoimmunity and lymphoproliferation, initiated equally early in life, with membranous glomerulonephritis, generalized enlargement of lymphoid organs, relapsing polychondritis and antiphospholipid syndrome. Autoreactive antibodies including ANA could be detected during laboratory investigation, while isohemagglutinins were absent. Important findings in the B-lymphocyte compartment were progressive reduction of CD19+ cells, impaired class switch, reduced numbers of memory B-cells, and increased CD21^low^ B-cells (Table 1).

**Table 1:** Clinical and laboratory characterization of the published patients with biallelic mutations in *PRKCD*

	Salzer, Santos-Valente et al.	Kuehn et al.	Belot, Kasher, Trotter et al.			Kiykim et al.
Patient/origin	P1/Turkey	P2/Mexico	P3/North Europe	P4/North Europe	P5/North Europe	P6 Turkey
Consanguinity	Yes	Probable	Yes	Yes	Yes	Yes
Disease onset/gender	1 year/male	3 years/male	10 years/female	3 years/female	6 years/male	3.5 yearss/male
Lymphoproliferation						
Hepato/splenomegaly	+	+	+	+	−	+
Lymphadenopathy	+	+	−	+	−	+
Autoimmunity						
Antiphospholipid syndrome	+	−	−	+	−	−
SLE-like skin manifestations	−	+	+	−	+	+
Kidney involvement	+	-	+	+	+	−
Other	Polychondritis	-	CNS	Autoimmune anemia		
Thrombocytopenia						
CNS vasculitis	−	−				
Other clinical features						
Recurrent infections	+	+	−	−	−	+
Autoantibodies						
ANA	+	+	+	+	+	+
Anti-dsDNA	+	+	−	−	+	−
Other	IgG anti-cardiolipin	Anti-RNP, Smith, SSA	−	−	−	−
Laboratory findings						
CD19+ B cells	Progressively ↑	↑	↓	↓	Normal	↑
Naïve B cells	Normal	↑	−	−	↑	↑
IgM memory B cells	↓	↑	−	−	↓ (CD19 + CD27+)	↓
IgG memory B cells	↓	↓	−	−	−	↓
IgG levels	Normal	↑	Normal	Normal	Normal	↑
IgM levels	↑	↑	Normal	Normal	Normal	Normal
IgA levels	Normal	Normal	Normal	Normal	Normal	Normal
NK cells number/function	Normal/−	Slightly ↓/↓	↓/−	Lower range/−	Normal	Normal/−
Lymph node histology						
	Reactive follicular hyperplasia with predominant B cells	Expansion of B cell areas and prominent B follicles	−	−	−	−
*PKCRD* mutation						
	c.1352 + 1G>A	c.1840C>T, p.Arg614Trp	c.1258G>A p.Gly510Ser	c.1258G>A p.Gly510Ser	c.1258G>A p.Gly510Ser	c.742G>A, p.Gly248Ser
Treatment						
	IVIg, anti-CD20 (2×)					
MMF§, corticosteroid	Corticosteroid					
Rapamycin	−	−	MMF	Hydroxychloroquine		
Outcome						
	Disease controlled	Excellent clinical response to therapy	Treating severe SLE manifestations	Died at age of 13 years	Clinical and laboratory remission	Clinical and laboratory remission

+: present. −: Not present, not performed or not mentioned in the manuscript. ↑: increased. ↓: decreased

*CNS* central nervous system

Since our initial description of PKCδ deficiency in 2013, five other patients (P2 to P6) from three unrelated kindred also bearing biallelic mutations in *PRKCD* have been published [19, 20, 58] (Table 1). The mutations described were located in the nuclear localization sequence of *PRKCD* (c.1840C>T; p.R614W; P2) [20], inside the activation loop (c.1258G>A; p.G510S; P3 to P5) [19] or within the regulatory domain (c.742G>A; p.G248S; P6) [58] (Fig. 1; Table 1). Similar to our patient, all those mutations led to absence or reduction of PKCδ expression.

The clinical manifestations of P2 were similar to our patient and he was diagnosed with an autoimmune lymphoproliferative syndrome (ALPS)-like disease [20]. In the same year, three PKCδ-deficient siblings (P3 to P5) diagnosed with SLE were reported presenting with lymphadenopathy, hepatosplenomegaly, nephritis, and arthritis [19]. In 2015, we and others described another patient (P6) with erythematous skin rash accompanied by fever and thrombocytopenia. Physical examination at that time point revealed partial alopecia, hyperpigmented skin rash predominantly in sun-exposed areas, cervical lymphadenopathy, hepatosplenomegaly, and mild hypotonia [58].

All six hitherto described patients showed symptoms before the age of 10 years and presented with hepatosplenomegaly, lymphoproliferation, autoreactive antibodies, and SLE or SLE-like autoimmunity. Lymphoproliferative features were seen in five patients from the four kindred (P1 to P4, P6), kidney involvement was present in four patients from two families (P1 and P3 to P5), and recurrent infections were observed in P1 and P2 (Table 1).

Laboratory investigations in all patients revealed the presence of autoantibodies and mostly unaltered T-cell numbers and function (Table 1). Only P2 presented with increased double-negative T cells. P2 also showed severely impaired NK-cell function, while NK cells from P6 demonstrated moderate decrease in cytolytic activity. Circulating B-lymphocyte counts were variable among the affected individuals, three out of six patients showing reduced or progressively reduced CD19+ cells (P1, P3, and P4). IgG memory B cells were reduced in the peripheral blood of all the patients, while predominant B-cell infiltration was apparent in peripheral lymphoid organs of two patients with lymphadenopathy and splenomegaly (P1 and P2). Four out of six PKCδ-deficient patients presented with nephritis (P1, P3-P5), and two patients had reduced levels of C3 and C4 (P3 and P4). Immunoglobulin levels were variable but three patients presented with elevated IgM levels (P1 and P2, P6) (Table 1).

Additional experiments demonstrated that cells bearing the *PRKCD* c.1352 + 1G>A or c.742C>A mutation showed reduced expression or activation of the PKC substrate MARCKS [59]. B cells from P1 (c.1352 + 1G>A) displayed increased *NF*-*IL6* and *IL*-*6* mRNA levels. B lymphocytes from P2 (c.1840C>T) showed slightly increased IL-6 and increased IL-10 levels. Decreased apoptosis and increased response to stimulation were demonstrated on B cells carrying *PRKCD* c.1840C>T or c.1258G>A mutation. Both of these Epstein-Barr virus immortalized lymphoblastic B-cell lines were characterized by B-cell hyperproliferative responses and resistance to PMA-induced or calcium-dependent apoptosis.

The phenotype resulting from autosomal recessive PKCδ-deficiency in humans expands findings obtained from mice related to the functions of the protein in B-cell development and activity [1]. A non-essential role for the protein in embryonic development or early survival had been suggested from mouse studies [1, 2]; however, presence of PKCδ is crucial for controlling B-cell expansion, considering that both human patients and mice presented with lymphadenopathy and/or hepatosplenomegaly [2, 19–21]. Moreover, increased apoptosis resistance of B cells derived from patients with mutations in *PRKCD* was demonstrated, when compared to wild-type cells [19, 20]. In human PKCδ deficiency as well as in *Prkcd*^−/−^ mice, B cells also produced increased amounts of the proinflammatory cytokine IL-6 compared to wild type, probably due to inhibition of NF-IL6 DNA binding activity [2, 20, 21]. Additionally, the production of autoreactive antibodies by plasma cells was significantly increased in mouse models [1, 2] and in all described patients [19–21].

The involvement of IL-6 in disturbed B-cell tolerance observed in patients with *PRKCD* mutation is supported by their phenotypical similarities with mice transgenically expressing human IL-6, including glomerulonephritis and enlargement of lymphoid organs due to massive B-lymphocyte proliferation [60]. Specifically, the uncontrolled B-cell proliferation observed in individuals with loss-of-function *PRKCD* mutations may however also relate to the already-mentioned decreased inhibition of IL-10 proinflammatory activity in the absence of functional PKCδ [57].

Although the impact of PKCδ deficiency in T-cell activation and proliferation could not be demonstrated for the patients described, and the influence of these mutations in NK-cell activity is yet to be understood, findings from mouse models expanded by human PKCδ-deficient patients validate the crucial role of PKCδ in controlling B-cell activation, differentiation, and apoptosis.

## PKCδ Deficiency as a Molecular Cause of Systemic Autoimmunity

The clinical phenotype of SLE is often variable, which suggests a heterogeneous pathophysiology of this disease [18]. To date, the etiology of SLE is still unclear; however, its pathogenesis has been associated with defective clearance of apoptotic cells, disturbed B- and T-cell activation, and cytokine-mediated inflammation. Clinically, almost half of the SLE patients present with malar rash and one third with nephritis (reviewed in [61]). The presence of anti-dsDNA antibodies is associated with higher risk of nephropathy and hemolytic anemia while presence of anti-cardiolipin antibodies and lupus anticoagulant are associated with thromboembolic events and miscarriage. Childhood-onset SLE patients, who are more likely to suffer from monogenic diseases, mainly present with severe nephropathy but also neurologic features, thrombocytopenia, and hemolytic anemia [62]. Clinically, typical features of nephritis, butterfly rash as well as anti-dsDNA antibodies demonstrated in the PKCδ-deficient patients are reminiscent of childhood-onset SLE [63].

Given the fact that PKCδ is crucial in the regulation of B-cell survival and apoptosis during B-cell development, the importance of intact B-cell signaling for peripheral tolerance is evident. Consistent with high concentrations of autoreactive antibodies, histology analyses of kidneys in *Prkcd*^−/−^ mice indicate glomerulonephritis with mesangial cell proliferation and deposition of IgG and complement component C3, similarly to the findings of nephritis and reduced levels of C3 and C4 in some of the PKCδ-deficient patients [19, 21].

Therefore, it is not surprising that also patients with common variable immunodeficiency (CVID) show increased susceptibility to immune dysregulation including autoimmunity, lymphoproliferation, and malignancies [64–68]. In CVID patients, autoimmunity is identified as the second most frequent manifestation after pneumonia [69], often closely associated with splenomegaly [69, 70]. Interestingly, the increased CD21^low^ B-cells and decreased memory B-cells displayed by PKCδ-deficient patients resemble findings of the subgroup CVID Ia from the Freiburg classification [71], which presents with impaired BCR-mediated calcium response [72] and shows higher frequency of splenomegaly and autoimmune cytopenias [71, 72].

The importance of this pathway in B-cell homeostasis could be further substantiated by the discovery of CVID-like B cell-deficient patients with or without autoimmunity carrying *PLCG2* mutations [67, 68]. PLCγ2 is an essential phospholipase downstream of the BCR upstream of PKCδ [63], catalyzing the conversion of 1-phosphatidyl-1D-myo-inositol 4,5-bisphosphate to 1D-myo-inositol 1,4,5-trisphosphate (IP3) and DAG using calcium as a cofactor [73].

Since PKCδ is required not only to regulate T-cell activation but also to intact B-cell signaling in the periphery and consequently to peripheral tolerance; disturbed activity of this kinase can most invariably prompt systemic autoimmune features as seen in other B-cell PIDs and in SLE.

## Concepts of SLE Therapy in Light of PKCδ Deficiency

Current treatment strategies in SLE are purely based on empirical observations. With the discovery of (mono)genetic defects underlying SLE pathology, different therapeutic options may come into place. Currently, standard treatments in SLE comprise hydroxychloroquine or other antimalarial agents; corticosteroids, which were also included in the treatment of P1 and P2 [20, 21]; and cytotoxic immunosuppressive drugs [74]. Interestingly, patient P6 showed amelioration of autoimmunity upon treatment using hydroxychloroquine [58]. The proposed molecular mechanism to justify the immunosuppression observed is an increase of CTLA4 expression, as demonstrated in LRBA-deficient patients treated with chloroquine [75].

Other effective drugs frequently used in such patients are mycophenolate mofetil (MMF) and rapamycin. P1 and P5 received MMF, and P2 was treated with rapamycin, all leading to disease control/clinical remission [19–21] (Table 1). Rapamycin is an inhibitor of mTOR, a central serine-threonine kinase for cellular metabolism, inflammation, and antigenic responses in several tissues [76], involved in the phosphorylation of the hydrophobic region of PKCδ [13].

Rituximab is a monoclonal antibody targeting the surface molecule CD20, expressed by mature B cells. It is effective in treatment of B-cell malignancies and rheumatoid arthritis, but the effects on SLE manifestations are still controversial, although approved in Europe and America for use as treatment of refractory patients [74]. Two courses of this drug were employed in P1; however, upon reoccurrence of peripheral blood B cells, autoantibody production was again detectable [21]. However, as rituximab targets all CD20-positive cells and does not influence selection of B cells, targeting of BAFF using belimumab would have been an interesting strategy, as BAFF signals are influenced by PKCδ and the survival factor adds to the selection process by altering apoptosis of transitional B cells.

Similarly to patients with active SLE [77], some patients with *PRKCD* mutation present with high levels of serum IL-6 and/or IL-10 [20, 21]. Tocilizumab is a humanized monoclonal antibody that prevents IL-6 binding and therefore blocks its proinflammatory functions. In our own experience on clinical treatment of patient P1, tocilizumab was efficient in reducing clinical and laboratory findings associated with autoimmunity; however, infectious complications prevented long-term use of therapy (unpublished results). Future studies will need to systematically assess blockade of IL-6 signaling as a therapeutic option in PKCδ deficiency.

Recent improvements in hematopoietic stem cell transplantation (HSCT) and gene therapy techniques have led to their increased use for potentially curative treatment of patients with severe PIDs [78–80]. Although clinical studies on HSCT in non-SCID are ongoing, it is still unclear which patients should undergo HSCT according to their phenotype/genotype, which conditioning regimen to choose, and how to manage those patients presenting with autoimmune diseases [79].

Recent advances in molecular diagnosis and treatment options therefore enable improved targeted therapies to be employed for the treatment of systemic autoimmunity in light of the identified molecular pathomechanism affected.

## Summary

The essential functions of PKCδ in B-cell homeostasis, in T-cell activation and proliferation, and more specifically in the autoimmune features observed in SLE were demonstrated in Prkcd-deficient mice (or mouse T-cells) leading to systemic autoimmunity. Recently identified human autosomal recessive PKCδ deficiency prompts severe symptoms including hepatosplenomegaly, lymphoproliferation, and SLE or SLE-like autoimmunity early in life. Such findings expand those of Prkcd-deficient mice in regard to the roles of this kinase in B-cell survival and apoptosis and implicate PKCδ and intact B-cell signaling in peripheral tolerance in humans. Taken together, the identification of human PKCδ deficiency provided proof-of-concept for monogenetic forms of early-onset SLE and allowed the identification of a key molecular pathomechanism relevant to generation of autoimmunity in humans.
